# Transcriptomic profiling with vascular tension analyses reveals molecular targets and phenotypes in preeclamptic placental vasculature

**DOI:** 10.3389/fendo.2024.1487549

**Published:** 2024-11-12

**Authors:** Eryun Zhang, Tao Zhou, Qiutong Zheng, Xiaomin Zheng, Yingying Zhang, Bailin Liu, Jiaqi Tang, Zhice Xu

**Affiliations:** ^1^ Wuxi Maternity and Child Health Care Hospital, Affiliated Women's Hospital of Jiangnan University, Wuxi, Jiangsu, China; ^2^ Obstetrics and Gynecology Hospital, Institute of Reproduction and Development, Fudan University, Shanghai, China; ^3^ Institute for Fetology, First Hospital of Soochow University, Suzhou, China

**Keywords:** placental micro-vessels, RNA-seq, *ELMO1*, preeclampsia, vascular tension

## Abstract

**Introduction:**

The placental vascular system plays an important role in the development of pregnancy hypertension in preeclampsia. The gene profiles of whole placental tissue (containing blood vessels and many other structural components) and pure vascular tissue should be very different. All previous reports using RNA-seq analysis in the placenta have tested its whole tissue or the villous part, and thus the gene profiles in the pure placental blood vessels are unknown.

**Methods:**

This study was the first to address this point with RNA-seq in human placenta at the transcript level. Isolated placental micro-vessels from normal and preeclamptic pregnancies were used for RNA-seq analysis, real-time quantitative polymerase chain reaction (RT-qPCR) verification, and vascular function tests. Furthermore, a vascular function-centric core network was constructed to show the gene-gene interactions and gene-function associations in the placental vessel system.

**Results:**

Differential expression analysis identified a total of 486 significantly changed transcripts. Bioinformatics analysis further confirmed that multiple genes were highly related to blood vessel and placental phenotypes. Several hub genes, including *ELMO1*, *YWHAE*, and *IL6ST*, were significantly reduced in the placental vessels in preeclampsia. Vascular tension experiments showed that angiotensin II-mediated vasoconstriction and exogenous NO donor sodium nitroprusside-induced vasodilation were decreased, while phenylephrine-mediated vascular responses were unchanged in placental micro-vessels in preeclampsia.

**Discussion:**

The results provide important insights into the pathological process in the placental vasculature in preeclampsia and offer great potential for further investigation of these molecular targets in the human placental vascular system.

## 1 Introduction

Preeclampsia (PE) is one of the most serious pregnancy complications with high morbidity and mortality for both the mother and baby (1). One major characteristic of PE is significantly increased blood pressure after the first 20 weeks of pregnancy. Interestingly, pregnancy hypertension is closely related to the development of the placenta. Notably, the majority of pregnancy-induced hypertension usually disappears after delivery of the placenta, strongly indicating a critical contribution of the placenta to the development of hypertension in pregnancy in PE (2).

It is well known that the placenta is rich in blood vessels. Most placental physiological functions, such as the support and maintenance of maternal-fetal-circulation for the delivery of oxygen, nutrients, and other substances between mother and fetus, are dependent on the vascular system in the placenta (3). Thus, anything that influences placental blood vessels or circulation, including structural and functional changes, would affect the health of both mother and fetus.

Regarding the development of hypertension and its underlying pathological process, it is also well known that the main basis for the development of hypertension, in general, may be related to alterations in either vascular structures or functions (4). Previous studies by us and others have demonstrated vascular dysfunction in response to various drugs in placental micro-vessels in PE (3). While exploring molecular cues to the underlying pathological mechanisms of PE, researchers have used RNA-seq to screen for differentially expressed (DE) genes in the placenta (5) and obtained important information on PE-related DE gene profiles in the human placenta. However, in addition to blood vessels, the placenta also contains a large amount of other non-vessel tissue. One of our research targets in the last two decades has been blood vessels (especially micro-vessels) in the placenta and umbilical cord (6). Thus, the initial question in this study was: can a DE gene profile generated by RNA-seq in whole placental tissue represent a profile for pure placental vessels? Obviously, the answer should be “No”, at least in precision medicine. To the best of our knowledge, there is no bioinformatics data or RNA-seq information on human placental micro-vessels. Therefore, this study was designed to be the first to use high-throughput sequencing technology to determine a DE gene profile in human placental blood vessels and examine transcript phenotypes in the placental vascular system in PE.

Considering that placental vascular dysfunction could be influenced by local inflammation or pro-inflammation, endocrine disorders, and pathological development of vascular structures (7), the bioinformatics analysis in the present study focused on DE genes related to these issues. Several endocrine factors or hormones, such as angiotensin II (Ang II), phenylephrine, and nitric oxide donor sodium nitroprusside (SNP) were employed in micro-vessel experiments to test for possible vascular functional changes in PE placentas.

## 2 Materials and methods

### 2.1 Patient selection

Healthy women with normal pregnancies (N=18) and women with preeclampsia (N=18) were recruited from the Wuxi Maternal and Child Health Hospital in China. The institutional ethics committee approved all procedures of this work (ref. no. 2022-01-0726-12), and all participants gave informed consent. Healthy pregnant participants were defined as having blood pressure not higher than 120/80 mmHg and no clinically significant complications and were the control group. Pregnant participants with preeclampsia were defined as having blood pressure>140/90 mmHg and significant proteinuria after 20 weeks of gestation. Women with essential hypertension or medical complications, such as diabetes, renal, or cardiovascular disease were excluded from the study. Human placentas were immediately acquired from the healthy and preeclamptic pregnant women after vaginal delivery or Caesarean section within 1 h. The placentas were preserved in an icy physiological solution. Human placental blood vessel branches were carefully isolated under a stereomicroscope. Blood vessel samples were gently and immediately separated from surrounding connective tissue and kept at -80°C before the experiments. The characteristics of the samples are shown in **Table 1**.

**Table 1:** Characteristics of the participants.

	Control (N = 18)	Preeclampsia (N = 18)
Age (year)	29.48 ± 4.75	29.76 ± 3.79
Gestational weeks	38.4 ± 1.25	36.54 ± 1.22^*^
BMI (kg/m^2^)	27.42 ± 2.66	27.22 ± 2.78
SBP (mmHg)	116.5 ± 3.81	148.50 ± 5.04^*^
DBP (mmHg)	74.5 ± 3.87	101.10 ± 5.02^*^

SBP, systolic blood pressure; DBP, diastolic blood pressure; BMI, body mass index. *P< 0.05.

### 2.2 RNA extraction

Approximately 50 mg of each placental vascular tissue sample was placed in grinding tubes with beads, and 1 mL of TRIzol reagent (Takara, Kusatsu, Shiga, Japan) was added to each tube and ground completely. Then, 0.2 mL of chloroform was added to each tube and shaken vigorously for 20 s. After the sample was left at room temperature for 5 min, the mixture was centrifuged at high speed (12,000 rpm, 4°C) for 15 min. The top phase was separated and mixed with 0.5 mL of isopropyl alcohol at room temperature for 10 min and centrifuged for 10 min (12,000 rpm, 4°C). The precipitate was washed with 1 mL of 75% ethanol, centrifuged for 5 min (7,500 rpm, 4°C), and the liquid was removed and dried at room temperature for 15 min. Then, 150 μL of diethyl pyrocarbonate (DEPC) H2O was added and gently mixed. Before library construction, all RNA samples were checked for quality: (1) RNA concentration, as well as purity, were tested, and the ratio of OD260/OD280 was 1.8~2.0 (Nanodrop); (2) RNA integrity and DNA contamination were checked using agarose gel electrophoresis; (3) The RNA integrity was assessed as a function of the RNA integrity number (RIN) >8.0, using an Agilent 2100 Bioanalyzer.

### 2.3 Construction of transcriptome libraries

Total RNA (1 μg) was used for the library preparation using a VAHTS Universal V8 RNA-seq Library Prep Kit for Illumina (Vazyme #NR605). The poly(A) mRNA isolation was performed using Oligo(dT) beads. The mRNA fragmentation was performed using divalent cations at high temperatures. Priming was performed using Random Primers. First-strand cDNA and the second-strand cDNA were synthesized. The purified double-stranded cDNA was then treated to repair both ends and add a dA-tailing in one reaction, followed by T-A ligation to add adaptors to both ends. Size selection of the adaptor-ligated DNA was then performed using DNA Clean Beads. The samples were then amplified by polymerase chain reaction (PCR) using P5 and P7 primers, and then PCR products were verified. Libraries with different indexes were multiplexed and loaded on an Illumina HiSeq instrument for sequencing using a 2x150 paired-end configuration according to the manufacturer’s instructions.

### 2.4 Sequencing data quality control

In order to remove technical sequences, including adapters, PCR primers, or fragments and quality of bases lower than 20, pass filter data in the fastq format were processed by Cutadapt (V1.9.1, phred cutoff: 20, error rate: 0.1, adapter overlap: 1bp, minimum length: 75, proportion of N:0.1) for high-quality clean data. Reference genome sequences and gene model annotation files of related species were downloaded from genome database websites, including National Center for Biotechnology Information (NCBI), University of California Santa Cruz (UCSC), and ENSEMBL. Hisat2 (v2.2.1) was then used to index the reference genome sequences. Finally, the clean data were aligned to the reference genome using Hisat2 (v2.2.1).

### 2.5 RNA-seq data analysis

The DESeq2 package was used to normalize raw expression counts, filter low-expressed genes, and calculate differentially expressed genes between different groups at the transcript level. The raw p values were further corrected [to generate false discovery rate (FDR) values] by using the Benjamini and Hochberg (BH) method. The combined strategy of FDR<0.05 and log2fc>1 (absolute fold change greater than two) was used to select significantly changed genes.

### 2.6 Bioinformatics analysis

To explore the functional potentials of differentially expressed genes, the ToppGene toolkit was used to perform gene list enrichment analyses (8). The embedded databases of phenotype associations, Gene Ontology (GO; including biological processes, cellular components, and molecular functions), and Reactome Pathways were used for functional enrichment. The full gene set of each functional database was used as background for the statistical calculations. Functional terms with p<0.05 were considered significantly enriched. The relationships between and among different genes were annotated using the STRING database (9).

### 2.7 Real-time quantitative polymerase chain reaction validation

Based on the results of the RNA-seq data analysis, the differentially expressed genes with both statistical significance and at least 2 or 3-fold difference were selected for further real-time quantitative polymerase chain reaction (RT-qPCR) validation. RNA was extracted using a previously described procedure (10). A first-strand cDNA synthesis kit (Takara, Cat#6210A) was used for the synthesis of cDNA strands from purified total RNA. A real-time PCR was performed with SYBR and analyzed on Quant Studio (Thermo Fisher Scientific) in a 96-well plate. The 2 ^−ΔΔCT^ method was used to comparatively quantify the mRNA levels of the target genes, and *ACTB* was used as an endogenous reference gene. All the primer sequences used in this study are listed in **Supplementary Table 1**.

### 2.8 Measurement of placental vessel tension

The placenta was preserved in ice-cold Krebs solution (containing NaHCO_3_ 25 mmol/L, NaCl 119 mmol/L, KH2PO_4_ 1.2 mmol/L, glucose 11 mmol/L, KCl 4.7 mmol/L, MgSO_4_ 1.0 mmol/L, and CaCl_2_ 2.5 mmol/L), and bubbled with 95% O_2_ and 5% CO_2_. Placental vessels (diameter: approximately 150 μm) were carefully isolated under a stereomicroscope. All blood vessel samples were gently and immediately isolated from the surrounding connective tissue and kept at 4°C in a HEPES solution (NaCl 115.0 mmol/L, KCl 4.7 mmol/L, MgSO_4_ 1.1 mmol/L, EDTA 0.51 mmol/L, CaCl_2_·2H_2_O 1.5 mmol/L, KH2PO_4_ 1.2 mmol/L, glucose 5.0 mmol/L, and HEPES 10.0 mmol/L, pH=7.4 with NaOH) for the experiments.

The human placental blood vessels were carefully cut into rings approximately 2 mm long under a stereomicroscope. An M4-myograph system (Danish Myotechniques, Aarhus, Denmark) was used to measure vascular tension in response to various drugs. A vascular ring of human placental micro-vessels (approximately 150 μm in diameter) was placed in a chamber of the system. The chamber was filled with HEPES solution and gassed with 95% O_2_ and 5% CO_2_. KCl (120 mmol/L) was used three times to induce optimal resting tension, which was treated as contraction normalization. After 60 min of equilibration, following serotonin (5-HT, 10^−4^mol/L)-mediated contractile platform, sodium nitroprusside (SNP, 10^−9^–10^−4^mol/L) was added into the solution with the vessel ring, and responses of vascular tone were continuously recorded. Cumulative concentrations of angiotensin II (AngII,10^−11^–10^−6^mol/L) or phenylephrine (10^−9^–10^−4^mol/L) were added to the chamber to test the vascular response curves. Each vascular ring was used for only a single drug test (SNP, Ang II, or phenylephrine only).

### 2.9 Statistical analysis

GraphPad 8.0 was used for statistical analysis. Data are expressed as mean ± standard error of the mean (SEM). A p-value < 0.05 was used to determine the statistical significance of the difference between the two groups using Student’s t-tests.

## 3 Results

### 3.1 Isolated placental vessels

**Figure 1:**
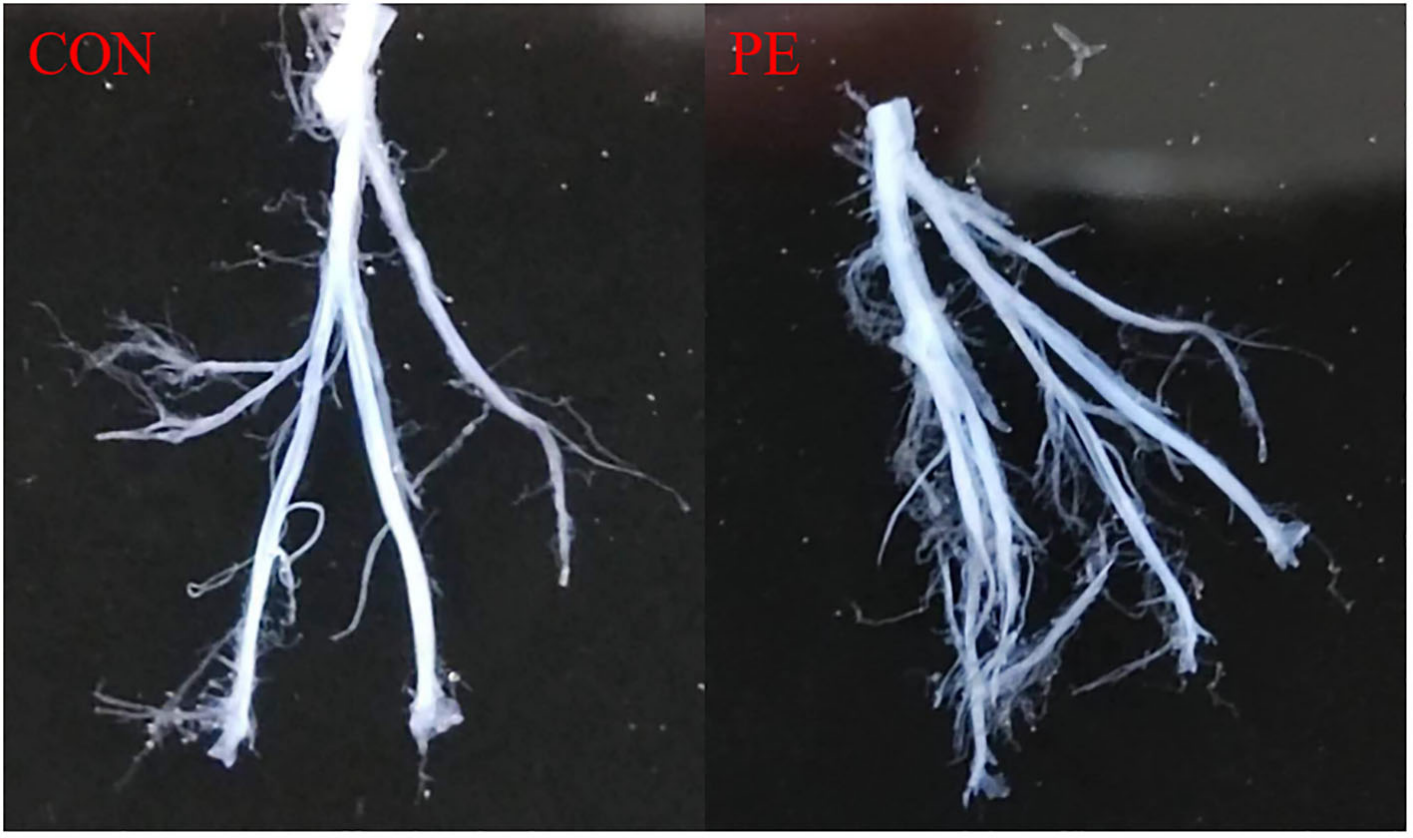
Placental blood vessels were separated from placental tissue. Con, control; PE, preeclampsia.

**Figure 1** shows a typical sample of human placental blood vessel structures which was isolated carefully under a stereomicroscope. All the placental vessel samples were separated from the surrounding tissue as shown in **Figure 1** and were used for both functional and molecular experiments, including RNA-seq, RT-qPCR tests, and vascular tension measurements.

### 3.2 RNA-seq analysis

**Figure 2:**
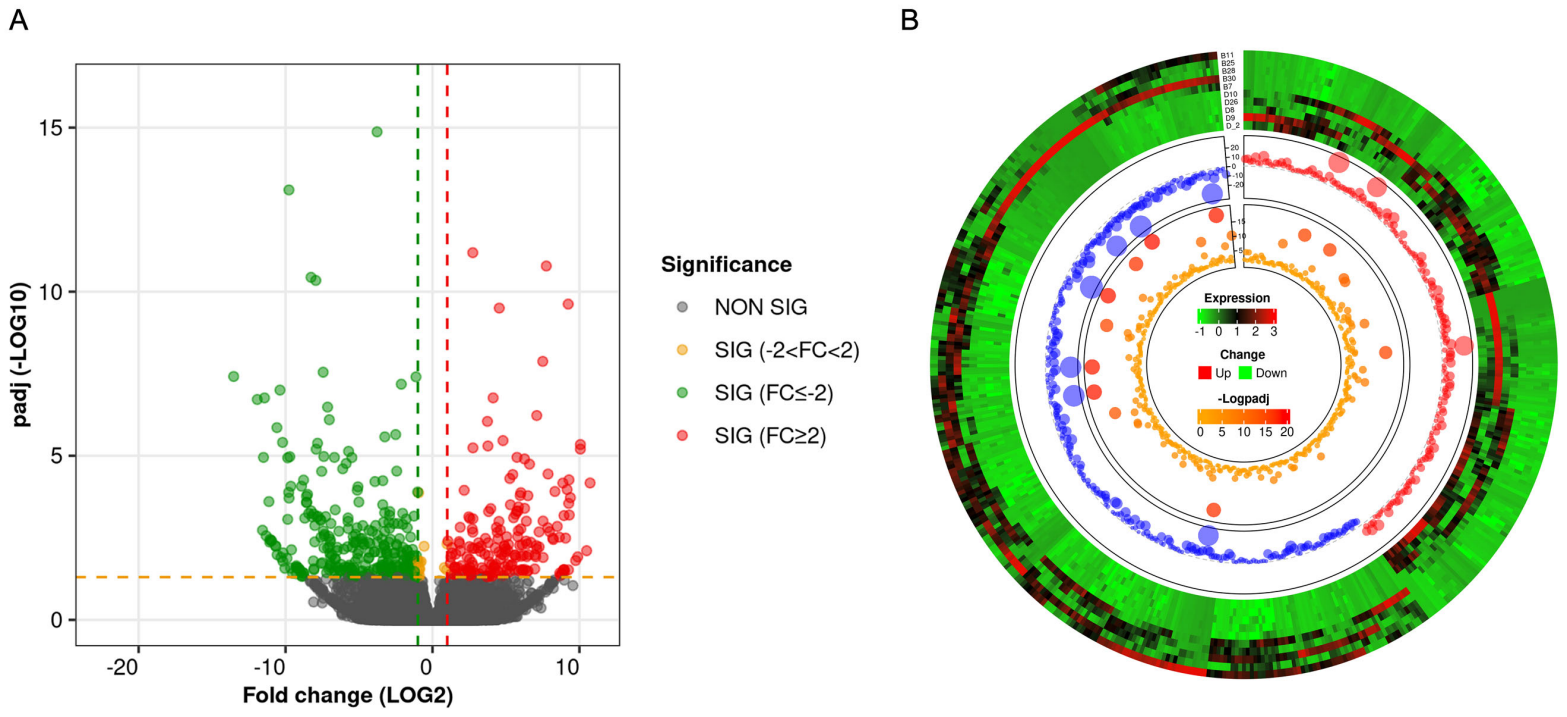
**(A)** Volcano plot for the comparison of the gene expression between different groups. **(B)** Circular heatmap of the differentially expressed transcripts.

RNA-seq analysis shows that there were a total of 62,710 transcripts in the human placental micro-vessel tissue. Comparative analysis demonstrated there were a total of 486 differentially expressed transcripts (products of protein-coding genes), with 197 upregulated and 289 downregulated in the PE group when compared to the control group. Bioinformatics analysis provided a heat map and volcano plot of differentially expressed transcripts in placental micro-vessel tissue between the control and PE groups (**Figure 2**). A detailed list of the differentially expressed transcripts is presented in **Supplementary Table 2**.

### 3.3 Bioinformatics analysis

**Figure 3:**
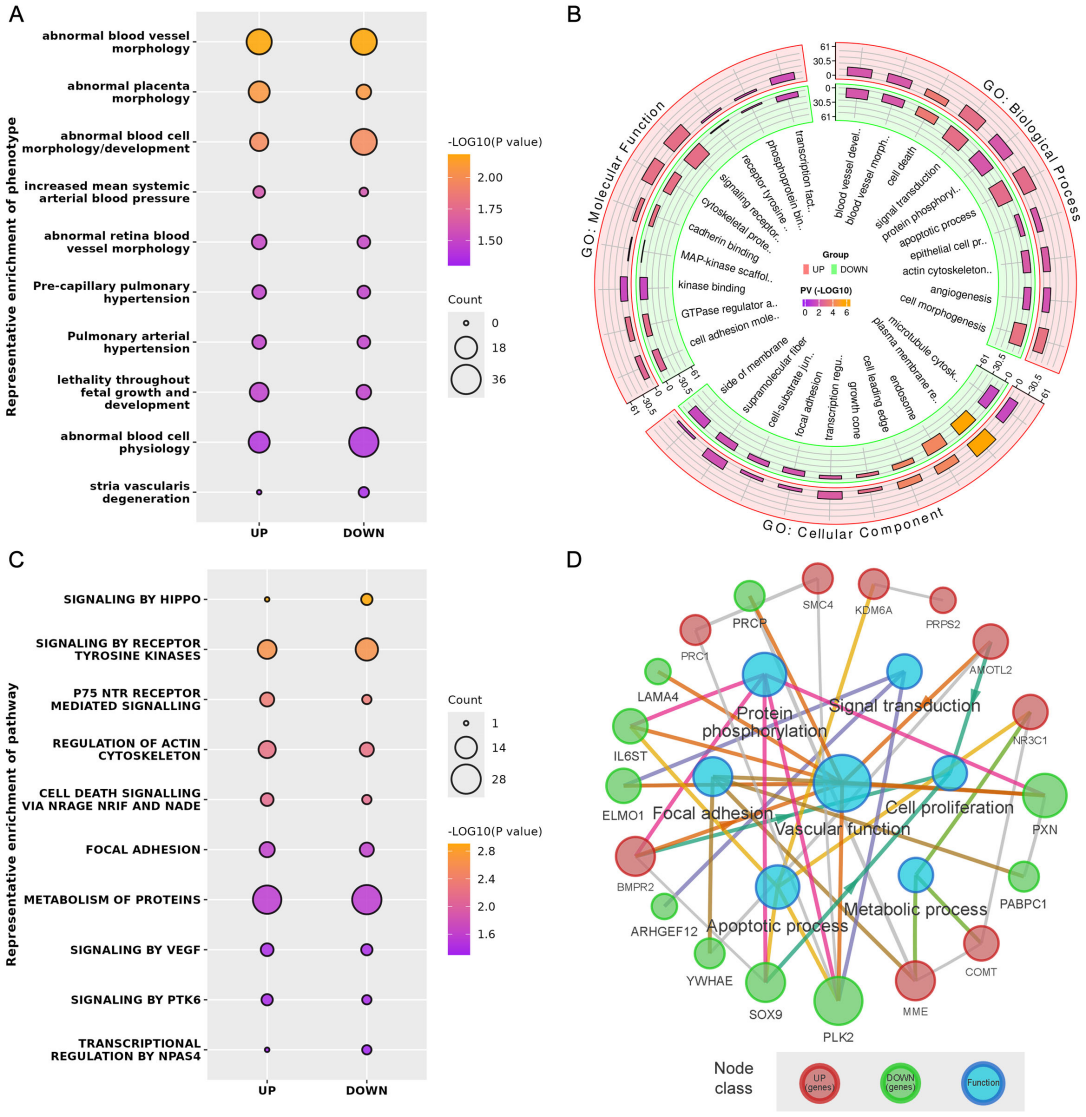
**(A)** Representative phenotype enrichment results. **(B)** Representative GO enrichment results. **(C)** Representative pathway enrichment results. **(D)** Vascular function-centric core network of the DE genes and major associated functions.

Phenotype enrichment analysis showed that the DE genes were highly related with blood vessel morphology, placental morphology, and hypertension (**Figure 3A**). GO and Reactome Pathway annotations further indicated the detailed functional associations at different levels (**Figures 3B, C**). Briefly, in addition to the predominant function of blood vessel development during pregnancy, these DE genes were also involved in the apoptotic process, cell proliferation, focal adhesion, transcriptional regulation, protein phosphorylation, and signal transduction (**Supplementary Table 3**). Considering both the gene-gene interactions and gene-function associations, a vascular function-centric core network was constructed (**Figure 3D**). These 19 hub genes were selected as potential key targets for understanding the pathological mechanism of preeclampsia in the human placenta (**Table 2**).

**Table 2:** Key hub genes identified in the vascular centric core network.

Gene name	Description	Change	log2FC	FDR
**COMT**	Catechol-O-methyltransferase	UP	6.26	1.25E-05
**PRC1**	Protein regulator of cytokinesis 1	UP	2.55	8.19E-04
**SMC4**	Structural maintenance of chromosomes 4	UP	1.88	1.51E-03
**PRPS2**	Phosphoribosyl pyrophosphate synthetase 2	UP	1.80	4.16E-03
**AMOTL2**	Angiomotin-like 2	UP	3.12	6.37E-03
**KDM6A**	Lysine demethylase 6A	UP	2.35	1.20E-02
**BMPR2**	Bone morphogenetic protein receptor type 2	UP	1.22	1.87E-02
**MME**	Membrane metallo-endopeptidase	UP	1.96	2.24E-02
**NR3C1**	Nuclear receptor subfamily 3 group C member 1	UP	1.52	2.51E-02
**PLK2**	Polo-like kinase 2	DOWN	-3.25	2.65E-06
**PXN**	Paxillin	DOWN	-3.33	1.07E-03
**YWHAE**	Tyrosine 3-monooxygenase/tryptophan 5-monooxygenase activation protein epsilon	DOWN	-1.74	7.41E-03
**IL6ST**	Interleukin 6 cytokine family signal transducer	DOWN	-2.52	1.30E-02
**ELMO1**	Engulfment and cell motility 1	DOWN	-3.08	1.82E-02
**SOX9**	SRY-box transcription factor 9	DOWN	-3.48	2.14E-02
**PRCP**	Proly lcarboxy peptidase	DOWN	-1.50	3.28E-02
**PABPC1**	Poly-A binding protein cytoplasmic 1	DOWN	-1.23	3.37E-02
**LAMA4**	Laminin subunit alpha 4	DOWN	-2.78	3.56E-02
**ARHGEF12**	Rho guanine nucleotide exchange factor 12	DOWN	-1.88	3.81E-02

### 3.4 Real-time quantitative polymerase chain reaction analysis

**Figure 4:**
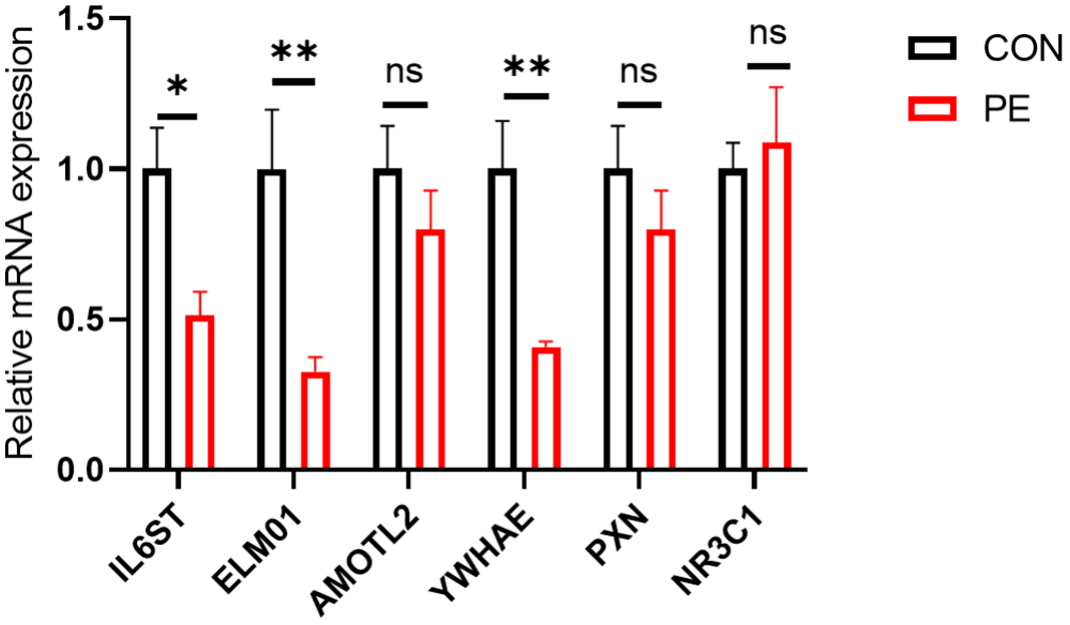
The results of further RT-qPCR validation. *P <0.05; **P <0.01; ns, no significance.

A total of six differentially expressed genes with at least 2-3-fold differences were used for further RT-qPCR validation. They are *IL6ST*, *ELMO1*, *AMOTL2*, *YWHAE*, *PXN*, and *NR3C1*. RT-qPCR analysis showed that half of these differentially expressed genes (*ELMO1*, *YWHAE*, and *IL6ST*) were significantly reduced in placental micro-vessel tissues in the PE group (**Figure 4**).

### 3.5 Micro-vessel tension mediated by drugs

**Figure 5:**
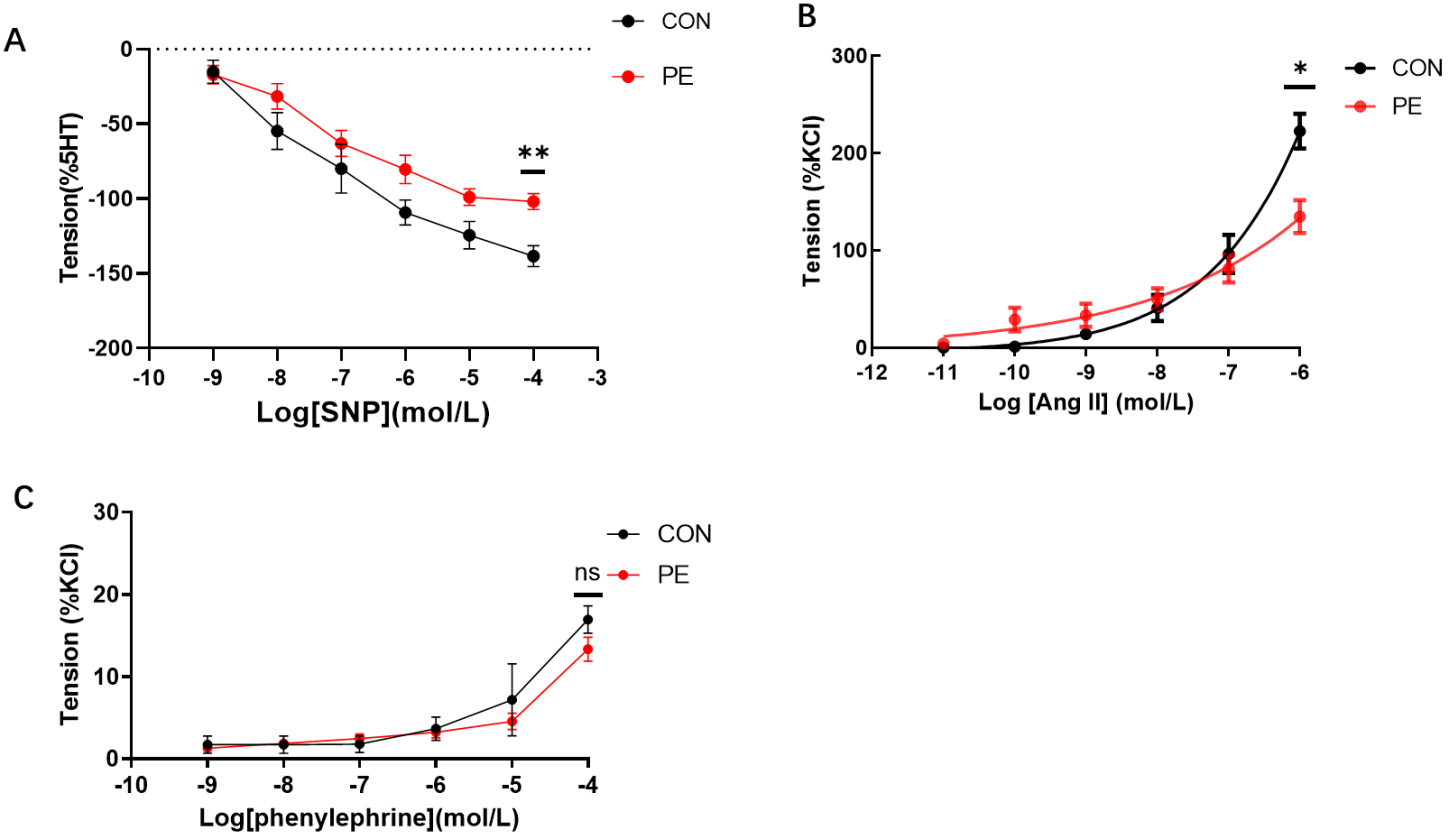
**(A)** The vasodilator agent sodium nitroprusside (SNP) mediated dilation responses in normal and preeclamptic human placental vessels. **(B, C)** Angiotensin II (Ang-II) and phenylephrine-mediated vascular responses in normal and preeclamptic placental micro-vessels. *P <0.05; **P <0.01; ns, no significance.

The functional experiments tested vascular tension responses to various drugs, that induced either vasodilation or vasoconstriction in placental micro-vessels. The accumulated concentrations of SNP-induced placental vascular relaxation were significantly reduced in the PE group (**Figure 5A**). This indicates that vascular dilatation capability was impaired in the placentas of the PE patients.

As **Figure 5B** shows, as the dose increased, Ang-II generated a significant dose-dependent vasoconstriction curve in both the control and PE groups, demonstrating the positive stimulating effects of Ang-II on human placental micro-vessels. Moreover, Ang-II-mediated placental vascular constriction was significantly reduced in the PE group (**Figure 5B**), indicating that the response of placental micro-vessels to Ang-II was damaged. Application of phenylephrine, another common vascular stimulator (11), to placental vascular samples in the chamber resulted in limited vascular tone in both the control and PE groups (**Figure 5C**). No significant change was observed between the control and PE placental vessels in response to accumulated concentrations of phenylephrine.

## 4 Discussion

The placenta plays a critical role in the maternal-placental-fetal circulation between the pregnant mother and her fetus. Anything wrong in a developing placenta may cause maternal diseases such as PE or a fetal growing issue such as intrauterine growth restriction (IUGR) (12). The human placenta is rich in micro-vessels, which are important for multiple functions. This study focused on placental micro-vessels under conditions of PE. RNA-seq analysis of the placenta is not novel (13). However, all previous analyses have tested whole placental tissue together with blood vessels and various surrounding tissues. In contrast, this study used separated placental micro-vessels after removing the surrounding tissues, as **Figure 1** shows. For example, a previous study showed a total of 1,950 differentially expressed mRNAs in the human placenta (whole tissue) in PE (14), much more than that in our study. This makes sense because their whole placental tissue not only contains vascular tissue but also other tissues. It is not strange that the molecular analysis results of whole placental tissue and pure blood vessel tissue could be very different. The present study was the first to use RNA-seq analysis to explore pure human placental micro-vessels in both healthy and PE groups. Therefore, all RNA-seq analysis-generated data regarding human placental micro-vessels in this study are novel and have not been published before.

Our transcriptomic analysis revealed a total of 486 genes in human placental micro-vessels that were differentially expressed due to PE (**Supplementary Table 4**). The heat map and volcano plot show the significantly upregulated or downregulated transcripts. Our new data demonstrate that all of these 486 up or downregulated genes were purely expressed in placental blood vessels, after excluding other placental tissues in PE. If one were to compare the genes between our results and previous studies, it would be found that some individual genes may be differentially expressed only in placental micro-vessels or surrounding tissues (15). This is an important contribution to further understanding the DE gene profiles in both whole placental tissues and micro-vessels related to PE. This also indicates which vascular DE genes may play a role in placental blood vessels linked to the development of PE.

Second, further bioinformatics analysis uncovered that certain differentially expressed genes are highly related to blood vessel morphology and hypertension, demonstrating novel phenotypes of DE genes in human placental vascular development in PE, which is characterized by increased blood pressure in the mid-late trimesters of gestation (16). Notably, many of the DE genes were shown to be not only closely related to blood vessel development and function but also linked with cell proliferation, focal adhesion, apoptosis, transcriptional regulation, signal transduction, and protein phosphorylation. To reveal the DE gene-gene interactions and gene expression-vascular function associations in the placental vessel system in the human placenta in PE, a vascular functional centric core network was constructed (**Figure 3D**). In theory, the 19 hub genes in the network could be considered and selected as potential key targets to further explore the pathological mechanisms of preeclampsia in the human placenta.

It is understood that it is inevitable that there are false positives or negatives in RNA-seq analysis, especially in human samples with high individual variation. Thus, RT-qPCR can be employed as an additional indicator to verify DE genes (17). In our experiments, among the six representative DE genes in placental vessels, the reliability of *ELMO1*, *YWHAE*, and *IL6ST* as DE genes in PE was further confirmed. Additionally, although *PXN* and *NR3C1* did not have significant p-values in the RT-qPCR, their change trends were consistent with the RNA-seq analysis.

The engulfment and cell motility protein (ELMO) is an evolutionarily conserved adaptor protein with three protein isoforms. *ELMO1* has been shown to be expressed in the zebrafish vasculature and has an important role in early developmental vascular processes (18). The present study was the first to demonstrate that *ELMO1* is also present in human placental vasculature, and may play an important role in the process of PE since it was confirmed as a reliable hub DE gene in the present study. The placenta is in a state of development alongside the growth of the fetus. The development of the vasculature is influenced by the angiogenesis process. Endothelial migration is a highly dynamic process and small GTPase is known to be a major regulator of the migration of endothelial cells during angiogenesis (19). Active GTPase interacts with *ELMO1* to regulate the actin cytoskeleton via the ELMO and DOCK pathways, which are involved in cellular migration (20)and the engulfment of apoptotic cells (21). Based on its temporal vascular expression, *ELMO1* is essential for the formation and function of the various blood vessels in zebrafish development (22). Our vascular functional experiments using the drug SNP demonstrated that endothelium-dependent vasodilatation in placental micro-vessels was damaged in the PE group, consistent with a previous report (23), while bioinformatics analysis and RT-qPCR validation revealed that the *ELMO1* transcript was significantly reduced in preeclamptic placental vessels. This association strongly indicates that altered *ELMO1* expression in the placental vasculature may contribute to PE-mediated placental vascular disorders, which eventually induce hypertension in PE cases. However, further studies are essential to clarify the action pathways for *ELMO1*-mediated pathological processes in the placental vascular system in PE.

In addition to *ELMO1*, *YWHAE* is another hub DE gene found in placental micro-vessels in PE. The YWHAE protein family is comprised of at least seven highly conserved subtypes of soluble acidic proteins. It can be widely combined with other proteins, including membrane receptors, kinases, phosphatases, and transcription factors (24), acting as an interaction protein bridge in a wide range of physiological and biological processes, such as cellular apoptosis, proliferation, metabolic regulation, and signal transduction (25, 26). To the best of our knowledge, there is no information regarding *YWHAE* as a DE gene in blood vessels, especially in the placental vasculature. Thus, it is very interesting that *YWHAE* was found to be differentially expressed and confirmed in PE placental vessels in the present study. Notably, Ang-II and SNP affect the vascular system via transmembrane receptors and PKC pathways with the involvement of kinases or phosphatases (27). Our vascular tension experiments showed that although phenylephrine-mediated vessel tone was unchanged between the control and PE placentas, Ang-II-induced vasoconstrictions were significantly decreased in placental micro-vessels in the PE group, demonstrating that Ang-II-mediated vascular regulation in the placental vasculature was damaged, while *YWHAE* expression was also significantly inhibited in the same placental vascular tissues.

Similar to *ELMO1* and *YWHAE*, *IL6ST* was also significantly downregulated in the PE placental vasculature in the present study. *IL6ST*, interleukin 6 cytokine family signal transducer, is an important member of a signaling axis with a prominent role in tumor growth, promoting cell survival, proliferation, migration, survival, and metastasis (28). Whether impaired expression of *IL6ST* in placental micro-vessels influences vascular cell biological processes and the observed vascular dysfunction in PE is worth further investigation. Moreover, all three DE genes (*ELMO1*, *YWHAE*, and *IL6ST*) have been demonstrated to be responsible for inflammatory signal reception (29–31) and anti-inflammatory responses in endothelial cells. Considering that one major pathological change in PE is placental inflammation (32), it appears that there is potential research value in further exploring these key DE genes and their functions in the human placental vasculature.

Finally, we should acknowledge the limitations of our study. We had to leave further in-depth and detailed studies, such as gene knockout and silence experiments to future investigations. These additional studies would be helpful to indicate a direct cause-result relationship, instead of an association connection, between the molecular and functional data in placental micro-vessels. In addition, because of budgetary and other reasons, including the fact that it is very difficult to obtain normal human non-placental vessels due to ethical concerns, non-placental vessel controls were not used in the present study. This study also focused on Asian women due to our limitations in obtaining non-Asian women’s samples; other ethnicities are also highly impacted by PE, but the target population of this study is only Asian women.

In conclusion, this study is the first to present a new expression profile of transcripts in human placental micro-vessels using high-throughput sequencing, revealing interesting DE gene phenotypes in preeclamptic placental vasculature. The finding of several hub genes that were reliably verified to have transcript alterations in the pure placental vessels, along with vascular functional changes, provide important data to further understand the pathological process in PE placentas, and offer great potential for further investigation of these molecular targets in the human placental vascular system.

## Data Availability

The original contributions presented in the study are included in the article/**Supplementary Material**. Further inquiries can be directed to the corresponding authors.
