# Supplementary material for: Transcriptomic profiling with vascular tension analyses reveals molecular targets and phenotypes in preeclamptic placental vasculature
Source: Front Endocrinol (Lausanne). 2024 Nov 12;15:1487549. doi: 10.3389/fendo.2024.1487549 (PMC11588436; doi:10.3389/fendo.2024.1487549)
Supplement: Supplementary file 1 [file Table1.docx]

Supplementary Material

# Supplementary Table 1

| **Primer Name** | **Primer Sequence** |
| --- | --- |
| IL6ST-F | CCACAAGCCAGCCAACAGTAAC |
| IL6ST-R | AACAAAGGAGCACATAGCCCAAAG |
| ELMO1-F | AACTCGCTTTCTCCATCTTGTA |
| ELMO1-R | CATCCGTCCAGATACAGTACTC |
| AMOTL2-F | GGAACAAGATGGACAGTGAAAT |
| AMOTL2-R | GCGATTTGCAGATTCCAATCTC |
| YWHAE-F | GGAAGGAGGCTGCGGAGAAC |
| YWHAE-R | CGAATAGGATGCGTTGGTGGAAG |
| PXN-F | CCTGACGAAAGAGAAGCCTAAG |
| PXN-R | CAGTTCATCCAAGAGACTCTCC |
| NR3C1-F | CTCCCATTCTGACCACCCTTCTC |
| NR3C1-R | ACCACCTTCCTGTCTCCTGTTTAC |

List of oligonucleotide primers used in this study
